# Impact of *Moringa oleifera* lam. Leaf powder supplementation versus nutritional counseling on the body mass index and immune response of HIV patients on antiretroviral therapy: a single-blind randomized control trial

**DOI:** 10.1186/s12906-017-1920-z

**Published:** 2017-08-22

**Authors:** Koy Tshingani, Philippe Donnen, Henri Mukumbi, Pierre Duez, Michèle Dramaix-Wilmet

**Affiliations:** 1 Higher Institute of the Medical Techniques of Kinshasa (ISTM), Kinshasa, Democratic Republic of the Congo; 2 Centre de Recherche en Épidémiologie, Biostatistiques and Recherche clinique, École de Santé publique/Université libre de Bruxelles (ULB), Route de Lennik n 808, CP 1070 Anderlecht, Brussels Belgium; 3 Centre de Recherche en Politique et Système de santé – Santé Internationale, Brussels, Belgium; 4 Actions Communautaires SIDA/Avenir meilleur pour les Orphelins en République Démocratique du Congo (ACS/AMOCONGO), Kinshasa, Democratic Republic of the Congo; 5 Department of Therapeutic Chemistry and Pharmacognosy, University of Mons, Mons, Belgium; 6 Centre de Recherche en Épidémiologie, Biostatistiques and Recherche clinique, École de Santé publique/Université libre de Bruxelles (ULB), Brussels, Belgium

**Keywords:** *Moringa oleifera*, Supplementation, HIV, Antiretroviral therapy, DR Congo, Body mass index, CD4 count

## Abstract

**Background:**

To achieve effective antiretroviral therapy (ART) outcomes, adherence to an antiretroviral regimen and a good immunometabolic response are essential. Food insecurity can act as a real barrier to adherence to both of these factors. Many people living with human immunodeficiency virus (PLHIV) treated with ART in the Democratic Republic of the Congo (DRC) are faced with nutritional challenges. A significant proportion are affected by under nutrition, which frequently leads to therapeutic failure. Some HIV care facilities recommend supplementation with *Moringa oleifera* (M.O.) Lam. leaf powder to combat marginal and major nutritional deficiencies. This study aims to assess the impact of M.O. Lam. leaf powder supplementation compared to nutritional counseling on the nutritional and immune status of PLHIV treated with ART.

**Methods:**

A single-blind randomized control trial was carried out from May to September 2013 at an outpatient clinic for HIV-infected patients in Kinshasa (DRC). Sixty adult patients who were at stable HIV/AIDS clinical staging 2, 3 or 4 according to the World Health Organization (WHO), and were undergoing ART were recruited.

After random allocation, 30 patients in the *Moringa* intervention group (MG) received the M.O. Lam. leaf powder daily over 6 months, and 30 in the control group (CG) received nutritional counseling over the same period.

Changes in the body mass index (BMI) were measured monthly and biological parameters were measured upon admission and at the end of the study for the patients in both groups.

**Results:**

The two study groups were similar in terms of long-term nutritional exposure, sociodemographic, socioeconomic, clinical, and biological features. At 6 months follow-up, patients in the MG exhibited a significantly greater increase in BMI and albumin levels than those in the CG. The interaction between the sociodemographic, clinical, and biological characteristics of patients in the two groups was not significant, with the exception of professional activity.

**Conclusions:**

Under medical supervision**,** M.O. Lam. leaf powder supplementation may represent a readily available and effective local solution to improve the nutritional intake and nutritional status of PLHIV undergoing ART.

**Trial registration:**

The study was retrospectively registered in the Pan African Clinical Trial Registry on 15 May 2015, no. PACTR201505001076143.

## Background

Many advances have been made over the past 30 years to decrease the impact of the human immunodeficiency virus (HIV/AIDS) including encouraging biomedical breakthroughs, particularly in terms of prevention and reduction of the risk of contamination. In recent years, the rate of new HIV infections, the risk of contracting comorbidities or AIDS-defining opportunistic illnesses, and the number of AIDS-related deaths have all fallen dramatically [1], and in many cases, virus replication can be sustainably controlled with antiretroviral therapy (ART) [2]. Nevertheless, additional challenges have emerged, notably *(i)* the persistence of latent reservoirs of HIV-infected cells in the organs of patients treated with ART; and *(ii)* onerous, lifelong ART, with side effects constraining adherence to therapeutic regimens [3].

The cascade of inflammatory reactions induced by HIV infection increases basal metabolism and general energy expenditure [4]. This causes a vulnerability to malnutrition for people living with HIV (PLHIV), particularly for those who are experiencing a decrease of appetite or metabolic disorders, or for those affected by food scarcity in limited resources setting [5]. With ART, the nutritional status often improves [6, 7] because of a good immunometabolic responsible to the effective ART outcomes [8]. For that reason, it is necessary to administer ART to patients in adequate nutrition conditions. It’s well known that at the untreated asymptomatic HIV clinical stage or symptomatic stage, an increase of the resting energy expenditure is observed [9, 10]. Therefore, an additional energy compensation (of 10, 20 or 30%, depending on the clinical HIV/AIDS staging) is required to maintain a stable nutritional status [7, 10, 11].

In both HIV-positive and HIV-negative people, the normal total daily energy intake from proteins and fat is 10–12% and 15–30%, respectively. A healthy diet should be rich in essential amino acids, as well as unsaturated rather than saturated fats. A diet able to cover the recommended daily intake (RDI) of micronutrients has been recommended to improve and sustain the health and nutrition status of HIV patients [12]. In the Democratic Republic of the Congo (DRC), inadequate energy intake and specific micronutrients deficiencies are prevalent among the general population [13]. Moreover, most PLHIV residing in the DRC present nutrient deficiency at ART inception [14–16].

Partners of the National Program for the Fight against AIDS (PNLS) in the DRC supply some malnourished HIV patients with industrial foods. Nevertheless, this supply is often limited in quantity and time. Some HIV care facilities in the DRC recommend the intake of *Moringa oleifera* Lam. (M.O.) leaf powder to improve the nutritional status of patients. This plant, belonging to the Moringaceae family, is recommended for several reasons: *(i)* it is dense in nutrients including unsaturated fatty acids [17], high-quality proteins (rich in essential amino acids), and micronutrients: minerals [17–19] and vitamins [20] compatible with the recommended dietary allowances. The high antioxidant activity of M.O. Lam. leaf powder extracts has been reported [21–23], as well as its ability to complement the calories of staple food in a limited-resource area and effectiveness as a supplement for PLHIV [18, 19, 24]*. (ii)* This plant is easily accessible and available to all for use as a nutritional supplement especially among PLHIV treated with ART [3, 11, 24]. The Congolese scientific community and general population support the use of this plant as a nutritional supplement [25].

The objective of this study was to evaluate the impact of M.O. Lam. leaf powder supplementation versus nutritional counseling on the body mass index and immune response of ART-treated patients in the DRC.

## Methods

### Type of study

A single-blind, randomized, and controlled trial was conducted from March to September 2013. This study was reviewed and approved by the ethics committee of the School for Public Health, University of Kinshasa, DRC, with reference ESP/CE/005/2010. The study was retrospectively registered in the Pan African Clinical Trial Registry with the reference number PACTR201505001076143.

### Participants and location of the study

HIV-infected patients treated with ART attending the outpatient clinic or referral ART center for PLHIV were invited to participate in this interventional study. All participants provided informed written consent for study participation, in compliance with the principles of the Helsinki Declaration II [26]. The aims as well as the procedures of the study were explained to the participants. Participants were informed of their right to withdraw from the study at any time. The anonymity of the participants was guaranteed and no personal details were recorded.

### Criteria for inclusion

Patients had to be 18 years or older; undergoing ART for at least 6 months; at clinical stages 2 and 3 or 4, according to the WHO clinical staging of HIV/AIDS [27]; and clinically stabilized (without opportunistic infections at inception of the study).

### Criteria for exclusion

Patients were excluded if they had any of the following: renal dysfunction, malabsorption problems or unstable weight in the 3 weeks and months preceding the beginning of the study, respectively. Pregnant patients and patients who did not consent or did not present at consultation were excluded. Data were collected at an outpatient center specialized for antiretroviral treatment, classified as a center of excellence at the national level and skilled in the care of PLHIV in Kinshasa, DRC, “*Action Comunautaire Sida et Avenir Meilleur pour les Orphelins en République Démocratique du Congo* (ACS/AMOCONGO)”.

## Intervention

### 1. Nutritional assessment

At the beginning of the study, a physical examination was performed and a blood sample obtained. The food frequency consumption of patients over the last 12 months was assessed using a validated Food Frequency Questionnaire (FFQ) in which ten Congolese foods were grouped according to a food composition table [28] and survey [29]. The collected answers made it possible to assess differences in food frequency consumption between the control and intervention groups. A trained nurse administered the FFQ to patients.

At the first and all subsequent consultations, nutritional status and clinical parameters were assessed. For the nutritional status, height (to the nearest centimeter), weight (to the nearest 0.1 kg), and mid-arm circumference (to the nearest 0.1 cm) were measured by two trained nurses. Methods were standardized before the study initiation to limit inter-observer variability. The following clinical data variables were collected: gastrointestinal problems, such as anorexia, asthenia, and inflammation of the oral mucosa; stomatitis, and the eventual presence of opportunistic infections.

Autonomy in terms of physical activity was assessed using a score to evaluate the self-reported activities, including leisure activities, gardening, home maintenance, housework, type of transportation used, and occupational activities (remunerated or not). Throughout the study period, patients were clinically assessed and anthropometric parameters were collected every month. Biochemical parameters were measured at inception and at the sixth month of the study.

### 2. Nutritional supplement

The M. O Lam. leaf powder was provided in bags of 100 g containing 35 g of protein, 69 g of fat, and less than 2 g of carbohydrates, providing 765 kcal **(**Table 1 **)**. Randomly assigned patients in the Moringa group (MG) were instructed to supplement their daily diet with 30 g of M. O Lam. leaf powder (or 3 tablespoons), equivalent to 232.5 kcal of energy. (An amount of 10 g of M. O Lam. leaf powder could be mixed with each of the three meals per day.) The control group (CG) received standardized dietary counseling designed to provide a healthy, balanced and energetic diet, so as to be able to maintain body weight.

**Table 1:** Nutrient content of M.O Lam. leaf powder

Nutrient	100 g	30 g [3tbsp^1^/daily] supplied	Recommended grams or percent of nutrient intake/day	Daily percent intake of recommendation supplied
Energy [kcal]	765	232.3	1800 ^2^	
Protein (g)^a^	35	10.5	10–12% ^3^	3
Fat (g)^b^	69	20.7	15–30% ^3^	8
Carbohydrate (g)^c^	<2	-	-	-
Soluble minerals^b^				
Calcium (mg)	2580	774	700–800 ^7^	87
Phosphorous (mg)	330	99	700 ^4^	14
Potassium (mg)	1820	546	4700 ^5^	11
Sodium (mg)	130	39	<2000 ^7^	20
Magnesium (mg)	310	93	220^7^	42
Trace elements				
Zinc (mg)	7	2.1	4.9–7 ^6^	30
Iron (mg)	26	7.8	9.3–19.6 ^7^	40
Vitamin A^d^(mg ≈ μg/RE)	145.6 mg ≈ 20.362	43.7 ≈ 6.11	270–300 ^7^	2

*RE*: retinol equivalents

^1^ Tablespoon

^a^Lab. of phamacognosia, food science and human nutrition at ULB,^b^ Lab. of agricultural analysis of the Liège provincial station

^c^Lab. of the Center for Applied Agronomic Research of Hainaut (CARAH),^d^ Lab. of the Federal Agency for Food Chain Supply Safety (AFSCA)

^2^ According to DRC, in FAO, Food and Nutrition in Numbers 2014, accessed: http://www.fao.org/3/a-i4175e.pdf

^3^ The value accepted for the safe level of intake is 0.75 g per kg/day

^4^ Institute of Medicine (2004), Dietary Reference Intakes for Water, Potassium, Sodium, Chloride, and Sulfate. Washington, DC: National Academy Press

^5^ Institute of Medicine (1997), Dietary Reference Intakes for Calcium, Phosphorous, Magnesium, Vitamin D, and Fluoride. Washington DC: National Academy Press

^6^ According to zinc moderate bioavailability diets

^7^ FAO/WHO Expert Consultation on Human Vitamin and Mineral Requirements (1998: Bangkok, Thailand). Vitamin and mineral requirements in human nutrition: report of a joint FAO/WHO expert consultation, Bangkok, Thailand, 21–30 September 1998, accessed December 2015: http://www.fao.org/3/a-y2809e.pdf

The GATHER counseling method [30] approved by WHO for the contraceptive methods choice was used in this study. Patients were received one after the other. A trained nurse was in charge of listening to patients’ nutritional problems and they were informed on better diets and practices. Patients were advised in simple terms to select a choice of foods based on personal preferences and financial resources to include in their diet. Each interview ended with a summary and an appointment for a check-up and monitoring.

The chosen diet was supposed to contain at least four groups of the following mandatory foods: *1.* The energetic foods group: roots and tubers (cassava, yams, potatoes, and taro), cereals (rice, maize), and plantains. *2.* Foods rich in protein: meat, fish, chicken, eggs, crustaceans, snails, caterpillars, insects, milk, cheese, yogurt, soy, beans, peanuts. *3.* Foods rich in vitamins and minerals: vegetables and fruits such as okra, tomatoes, carrots, spinach, squash, green beans, cabbage, bananas, pineapple, mango, papaya, oranges, tangerines, mangosteen, grapefruit, apples, grapes, amaranth, cassava leaves. *4.* Use of best practice should be followed: food must be diversified and contain at least one food from each group in each meal; leftover food should be kept in hygienic conditions; hygienic practices should be observed throughout the preparation and cooking process; drinkable water should be used; no alcohol and tobacco consumption.

A month’s supply of M.O. Lam. leaf powder was provided to each patient in the MG. Compliance with the supplement regimen was checked by questioning patients during their monthly visits to the service. Medication including ART was supplied to patients in both groups according to Congolese national ART guidelines by the pharmacist immediately after the medical consultation and before the nutritional intervention that took place at the HIV outpatient center. The pharmacist offered advice regarding compliance to ART for all patients, regardless of the study group.

### Biochemical markers

Blood samples were collected twice during the study, at inception and after 6 months, for both study groups. Hemoglobin was evaluated by the hematocrit assay method; an enzymatic assay quantitatively determined the levels of albumin and creatinine; and a flow cytometric measurement was used for CD4 lymphocyte counts. These analyses were carried out at ACS/AMOCONGO laboratory, approved by the PNLS. A semi-quantitative method was used for analysis of CRP- reactive protein at the laboratory of the National Institute of Biomedical Research (INRB) of Kinshasa, DRC. The Abbott Real-time assay to quantify HIV-1 viral load was carried out at the national reference laboratory for the PNLS & STIs program, Kinshasa, DRC.

### Characteristics of the M.O. Lam. Leaf powder

M.O. Lam. leaf powder was derived from “M.O. Lam. plant,” Congolese ecotype. This plant was authenticated in the DRC in 1941 at Mongala in the Equateur province by Mr. Germain. A specimen is stored in the herbarium INERA collection at the Faculty of Sciences of the University of Kinshasa (UNIKIN) with the reference GERMAIN n°1064. The M.O. Lam. leaf powder was manufactured, processed, and packaged in the setting of the horticultural program of the Presbyterian community of Kinshasa, DRC.

### Nutritional properties of M.O. Lam. Leaves powder

The M.O. Lam. leaf powder Congolese ecotype contained, on average, 35.0 g of protein in 100 g of M.O. Lam. leaf powder. The total fat content (triglycerides, fatty acids, and alcohol glycerol) was 69 g and the carbohydrate content was less than 2 g per 100 g of M.O. Lam. leaf powder **(**Table 1). The levels of soluble minerals and trace elements are also shown in Table 1. A number of unsaturated and saturated fatty acids, including mono and polyunsaturated fatty acids, have been listed; their proportions were as follows: myristate 5%, palmitate 36%, stearate 6%, oleate 16%, linoleate 18%, linolenate 2%, and non-identified components 17%. The major phytosterols and alcohols were also identified: 1-eicosanol 1.3%, 1-tetracosanol 3.4%, 5-alpha-cholestane 6.8%, 1-hexacosanol 0.8%, 1-octacosanol 4.2%, cholesterol 1.3%, campesterol 3.3%, 1-triacontanol 15.2%, β-sitosterol 12.2%, stigmasterol 22.0%, and non-identified components 29.5%.

## Dosage methods of the nutrients contents in the M. O lam. Leaf powder

### Preparation of the M.O. Lam. Leaf powder

fresh M.O. Lam. leaves were collected and washed, then rinsed under flowing water and allowed to drain for approximately half an hour. The leaves were dried in the absence of direct sunlight in a sealed room for three to 4 days until fully dried.

### Measurement of total protein content

The total protein content was determined using the Digesdahl digestion apparatus method [31], which produces a mineralized product for the determination of total Kjeldahl nitrogen. The percentage of protein was calculated assuming that 1 g of nitrogen is equivalent to 6.25 g protein.

### Measurement of total fat content

The amount of fat was determined by the Soxhlet method: an extraction with warm hexane was performed and the product weighed before extraction and after processing in an oven at 75 °C.

## Profiling of the fatty acid, fatty alcohols, and phytosterols

The GC profiling of fatty acids was performed by a protocol adapted from Ichihara et al. [32] on a Shimadzu GC17A gas chromatograph equipped with a flame ionization detector (FID). The samples were analyzed on a FFAP CB fused-silica capillary column, 25 m × 0.32 mm i.d. (Chrompack, the Netherlands). Helium was used as the gas vector (2.1 mL/min). The injector and detector were maintained at 250 °C. Column temperature was initially kept at 80 °C for 1 min, then gradually increased to 230 °C at 8 °C/min rate and held for 6 min at 230 °C. About 150 mg of oil were transferred to a 25 mL volumetric flask, dissolved in n-hexane and filled to volume; 2.5 mL of this solution was transferred to a Teflon-capped derivatization vial, 2.5 mL of a 2 mg/mL hexane solution of methyl heptadecanoate (internal standard) added, and evaporated to dryness under nitrogen. 2.5 mL of 0.5 M hydrochloric acid in methanol were added, the flask capped, and the contents sonicated for 5 min with heating to 80 °C for 30 min. After cooling and the addition of 2.5 mL 0.5 M sodium hydroxide and 500 mg sodium chloride, the mixture was extracted with 10 mL of n-hexane. Chromatographic peaks were identified by comparison with retention times of methylated fatty acids standards.

The GC profiling of fatty alcohols and sterols was performed using a protocol adapted from the European Pharmacopoeia [33] on a Shimadzu GC17A gas chromatograph equipped with a flame ionization detector (FID). The samples were analyzed on a CP-Sil-5 CB fused-silica capillary column, 25 m × 0.32 mm i.d. (Chrompack, the Netherlands). Helium was used as the gas vector (2.0 mL/min). Injector and detector were maintained at 340 °C. Column temperature was initially kept at 200 °C for 1 min, then gradually increased to 325 °C at 8 °C/min rate and held for 20 min at 325 °C. About 1.5 mL were transferred to a round-bottomed flask and 25 mL 2 M potassium hydroxyde in water-ethanol (50:50) added. The solution was re-fluxed for 30 min and cooled; 25 ml water was added and the solution transferred with rinsing into a separating funnel. The solution was extracted with 50, 40, and 30 mL diethyl ether; the combined extracts were washed with water until neutral reaction, evaporated to dryness, and taken up in 10 mL of diethyl ether. Two mL of this solution were transferred in a Teflon-capped derivatization vial and evaporated to dryness under nitrogen. One mL 1 mg/mL cholestane (internal standard) in a silylating mixture (Sigma-Aldrich, silylating mixture I according to Sweeley) was added and the solution left at room temperature for 90 min.

## Measurement of mineral components

The sample was incinerated at 500 °C, and then the ash was dissolved with HCl and filtered. An atomic absorption spectrometry method based on ISO (the International Organization for Standardization) 6869, was used for the determination of major minerals after acid mineralization of the sample ash. Phosphorus was analyzed based on a colorimetry method derived from ISO (the International Organization for Standardization) 6869.

## Main parameters of outcomes assessment

### Primary parameters

The primary outcomes assessed were changes in the body mass index and biochemical markers after 6 months of follow up. Body mass index was calculated by the ratio of weight in kilograms to height in square meters. For assessment of the impact of supplementation on BMI changes, the BMI was assessed monthly during 6 months, and the evolution compared between the sixth month and study admission for both groups. The evolution of biochemical markers was assessed by comparing the patients’ biochemical marker levels at sixth months with those at inception.

### Secondary parameters

The secondary outcomes assessed were the evolution of BMI of patients in both study groups according to their sociodemographic, socioeconomic, clinical, and biological characteristics during the follow-up period and from inception to 6 months. Sociodemographic and socioeconomic data were collected upon inception of the study. They included: age, marital status, living environment, household size, level of education, and professional activity. These variables were categorized as age ≤ or >50 years old, according to the literature [34]; dichotomized marital status: married versus other civil status – grouped as divorced, widow(er) or single. Living environment was classified as living with others (e.g. a parent) or living alone. The household size was classified as no children, 1–3 children, 4 or more children; level of education was classified as low level (had not completed secondary education) or high level (completed secondary education or above). The professional occupation of patients was dichotomized into patients with or without professional activity. The number of years on ART was sought at inception and was dichotomized to patients as < or ≥ than 5 years on ART. The WHO clinical staging of HIV/AIDS was divided into an early stage, encompassing stages 1 and 2, and an advanced stage, including stages 3 and 4.

### Sample size

The sample size was calculated taking into account the standard deviation (SD) of a 3.3 kg/m^2^ gain in BMI observed in a cohort study of PLHIV on ART after a six-month follow-up [35], assuming there was at least a 2.5 kg/m^2^ observed gain in BMI difference between the patients in the intervention group and those in the control group. 80% of power of test was estimated with a type I error of 5%. The sample size was estimated to be 60 patients, 30 for each study group.

### Randomization

Healthcare providers identified eligible patients during consultation in their office and enrolled them to the study after obtaining the informed consent and maintaining ethical standards. Eligible patients were referred to the HIV-outpatient service where they were allocated into study groups based on numbers randomly generated by the Excel standard program: even numbers assigned patients to the MG and odd numbers assigned patients to the CG. The allocation of patients to the study groups was carried out by the principal investigator. The social workers, who had previously been trained, were responsible for the supply of M. O Lam. leaf powder and dietary counseling. Healthcare providers, including doctors, nurses, and a pharmacist, as well as the laboratory technicians were blinded to the allocation of patients to the study groups.

### Data analysis

Hemoglobin was dichotomized as <12 versus ≥12 g/dl, albumin was dichotomized as <3.5 versus ≥ 3.5 g/dl, blood creatinine was dichotomized as <1.4 versus ≥1.4 g/dl in men and <1.2 versus ≥1.2 g/dl in women. C-reactive protein was categorized into <6 versus ≥6 mg/l. For viral load, the number of copies/ml was dichotomized to undetectable viral load for patients with less than 40 copies/ml (<1.6 log) and detectable for those with more than 40 copies/ml (≥1.6 log) [36]. The CD4 count was dichotomized to <350 versus ≥350 cells/l. To appreciate the rate of virology failure among the patients followed in our study, the viral load parameter was dichotomized to <1000 copies/ml versus ≥1000 copies/ml.

Pearson’s chi-squared test, Student’s t-test, and the Mann-Whitney test were used to compare the distributions, the averages, and the medians, respectively. Analyses of variance for repeated measurements were performed to compare the change in nutritional status from inception to the sixth month between the two study groups. Multiple linear regressions were performed to explore the interaction effects between the sociodemographic, socioeconomic, and clinical factors on BMI gain at 6 months between the study groups. The Statistical Package for Social Sciences (SPSS) version 22 software and Open Epi version 2.3.1 were used.

## Results

### Participant flow

**Fig. 1:**
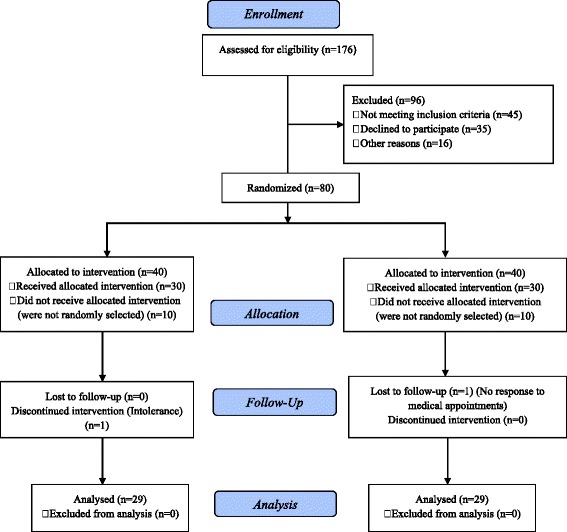
Flow diagram of participant progress in both study groups

During the study period, 176 patients from the outpatient clinic or ART reference center in Kinshasa, DRC, were eligible for the study. Ninety-six were excluded for not fulfilling the inclusion criteria or declined to participate. The randomization of the 80 remaining patients was performed independently by healthcare providers; 60 patients were randomly selected among those who consented to participate to the study on March 22, 2013. All the patients were informed of the potential merit of nutrition support. Thirty patients were randomly selected and allocated to the group receiving M.O. Lam. leaf powder (MG) supplementation and 30 patients were randomly allocated to the control group receiving nutritional counseling (CG). Among the 30 patients allocated in the MG, one patient did not tolerate the M.O. Lam. leaf powder, lost all motivation and stopped taking it. One patient in the CG dropped out of the study. Fifty-eight patients completed the 6 month follow-up (29 for each study group) (Fig. 1).

### Characteristics of the patients upon admission

The patients in the MG were similar to those in the CG in terms of sociodemographic, socioeconomic, and clinical aspects. Females were predominant in both groups; there were few married people in either group, and only a few patients did not have children. The BMI means for the MG and CG were 21.8 (SD 4.2) and 21.4 (SD 3.8) kg/m^2^, respectively. Patients at stages 3 or 4 of AIDS, according to the WHO clinical staging, but who were stable or asymptomatic were prevalent in both groups. The median duration of follow-up for ART was 4.5 (min–max 1–8) and 5.5 (min–max 0–14) years for the MG and CG groups, respectively; the patients mainly received first-line ART regimen (80 versus 63%, respectively) **(**Table 2).

**Table 2:** Sociodemographic, economic, and clinical characteristics of patients upon admission to the study

Variables	Moringa group Median (min–max)		Control group Median (min–max)		*P*
Gender					
Male		24		23	0.99
Female		76		77	
Age (years)	49.5 (21–64)		47.0 (18–61)		0.45
18–49		50		70	0.78
50–64		50		30	
Marital status					
Married		33		30	0.11
Others		67		70	
Living Environment					
At home		47		67	0.12
With third party		53		33	
Household size					
No children		10		13	0.91
≤3 children		43		43	
≥4 children		47		43	
Educational level					
Low		60		80	0.09
High		40		20	
Professional activity					
Not engaged		37		43	0.59
Engaged		63		57	
Anthropometric parameters					
Height (cm)	164.0 (6.0)		163.1 (7.9)		0.55
Weight (kg)	59.6 (12.9)		56.9 (10.8)		0.29
BMI (kg/m^2^)	21.8 4.2)		21.4 (3.8)		0.99
<18.5		23		20	0.75
≥18.5		77		80	
WHO Clinical staging					
Stage 2		17		27	0.34
Stage 3 & 4		83		73	
Duration of ART (years)	4.5 (1–8)		5.5 (0–14)		0.49
<5		70		50	0.11
≥5		30		50	
ART regimen*					
AZT + 3TC + NVP		80		63	0.32
TDF + 3TC+ LPV/r		17		33	
ABC + DDI + LPV/r		3		3	

*ART* antiretroviral therapy, *AZT* Zidovudine, *3TC* Lamivudine; *NVP*, Nevirapine, *TDF*, Tenofovir; *LPV/r* Lopinavir +Ritonavir, *ABC* Abacavir, *DDI*, *Didanosine*

At baseline, most of the biological biomarkers of patients were statistically comparable between the MG and CG. However, the mean albumin level and the proportion of pathological CRP level of patients in the MG were significantly lower than those in the CG **(**Table 3 **)**.

**Table 3:** Biological characteristics of patients upon admission to the study

Variables	*n* = 30	Moringa group	%	*n* = 30	Control group	%	*P*
		Median (min–max)			Median (min–max)		
		Mean (SD)			Mean (SD)		
Hb (g/dl)		12.2 (1.3)			12.3 (1.4)		0.99
<12			37			40	0.79
≥12			63			60	
CD4 (Cells/μl)		432.4 (195.0)			459.4 (215.0)		0.61
<350			30			30	0.99
≥350			70			70	
VL(copies RNA/ml)							
<40 (undetectable)			70			67	0.78
≥40 (detectable)			30			33	
Log10							
<1.6 (undetectable)			70			63	0.58
≥1.6 (detectable)			30			37	
VL (copies/ml)							
<1000			97			87	0.23
≥1000			3			13	
Albumin (g/dl)		3.3 (0.8)			3.9 (0.7)		0.018
<3.5			57			30	0.03
≥3.5			43			70	
CRP (mg/l)							
<6 (normal)			83			60	0.04
≥6 (pathological)			17			40	
Creatinine (g/dl)							
Men	7			7			
≤1.4			100			100	-
>1.4			0			0	
Women	23			23			
≤1.2			100			100	-
>1.2			0			0	

*Hb* Hemoglobin, *CD4* lymphocytes cells count, *VL* viral load, *CRP* C-reactive protein, *SD* standard deviation

### Changes of the anthropometric and biological characteristics during the follow-up

After 6 months of follow-up, patients in the MG showed a significant increase in BMI and in albumin compared to those in the CG. BMI mean [IC in 95%] increase was 1.9 [−0.5 to 3.7] kg/m^2^ in the third month, and 2.0 [−0.2 to 4.0] kg/m^2^ in the sixth month in the MG compared to the mean in the CG (*P* < 0.001) (Table 4). We observed significant changes in the mean albumin level of MG patients from 3.3 [SD 0.9] g/dl at baseline to 3.9 [SD 0.8] g/dl 6 months later while the mean albumin level of patients in the CG remained the same (3.9 [SD 0.7] g/dl) (*P* = 0.012) **(**Table 5 **)**. However, the mean CD4 lymphocyte counts at 6 months in comparison to the baseline were not significantly different between the two groups **(**Table 5 **)**. They were 51.3 (100.0) cells/μl for the MG and 63.9 (107.3) cells/μl for the CG (*P* = 0.64).

**Table 4:** Changes in the body mass index (BMI) of patients in the two study groups during the follow-up

Study groups	Mean BMI (SD)							
	n	Admission	1st month	2nd month	3rd month	4th month	5th month	6th month
Moringa group	30	21.8 (4.2)	22.9 (4.2)	23.5 (4.1)	23.8 (4.0)	24.1 (4.0)	24.1 (4.2)	24.4 (4.0)
Control group	30	21.4 (3.8)	21.6 (3.8)	21.7 (3.7)	21.9 (3.7)	22.1 (3.6)	22.3 (3.7)	22.4 (3.7)
Diff (CI at 95%)		0.4 (−1.3 to 2.7)	1.3 (−1,1 to 3.1)	1.8 (−0,9 to 3.3)	1.9 (−0.5 to 3.6)	2 (−0.3 to 3.7)	1.8 (−0.2 to 3.7)	2 (−0.2 to 4.0)
P (interaction)								<0.001

*Diff [CI at 95%]* Difference [confidence interval at 95%], *SD* standard deviation

**Table 5:** Changes in serum albumin and comparison of average of delta CD4 counts (SD) for the two study groups between admission and sixth months

Studies groups		Admission	6th month		*P*
		Mean (SD)	Mean (SD)		
Moringa group	28	3.3 (0.9)	3.9 (0.8)		
Control group	29	3.9 (0.8)	3.9 (0.7)		
Diff. [CI at 95%]		0.6 (0.15–1.0)	0 (−0.4–0.4)		
P = Interaction*					0.012*
		Mean CD4 (SD)	Mean CD4 (SD)	Mean Delta CD4 (SD)	
Moringa group	28	420.0 (195.0)	471.3 (202.0)	51.3 (100.0)	
Control group	29	467.8 (214.6)	531.7 (193.4)	63.9 (107.3)	
P = t-student**					0.64**

P* = Interaction, P** = t-student

Although the patient BMI gains were different at 6 months between the two study groups, these differences were not statistically significant according to the sociodemographic, clinical, and biological characteristics of patients (i.e. the interaction between the study groups and age, sex, duration of follow-up under ART, CD4 lymphocyte counts, and viral load of patients was not significant), with the exception of the BMI gain observed among patients when stratified for professional activity. Indeed, after adjustment for age, sex, duration of follow-up under ARTs, CD4 cell count, and viral load, we observed 1.95 kg/m^2^ further gain in patients with professional activity who received supplementation with M.O. Lam. leaf powder compared to those received nutritional counseling. (Table 6). Whereas in patients without professional activity, the BMI gain was greater in the intervention group, the difference between the two study groups was smaller (0.64). At the end of the study, we did not observe significant differences in the proportion of patients presenting elevated creatinine levels, or viral loads (HIV-RNA-1) that were higher than 1000 copies/μl between the study groups **(**Table 7 **)**.

**Table 6:** Increase of BMI at sixth month in study groups according to professional activity (PA), adjusted for sociodemographic, socioeconomic, clinical and biological characteristics of the patients

Variable	BE (ES)	*P*
Patients with PA		
Control group	1.06 (0.26)	
Intervention group	3.01 (0.38)	
Difference (I-C)	+1.95 (0.42)	<0.001
Patients without PA		
Control group	1.01 (0.28)	
Intervention group	1.65 (0.47)	
Difference (I-C)	+0.64 (0.48)	0.194

Adjusted by Age, sex, duration under ART, CD4 lymphocyte counts, viral load

Interaction: *P* = 0.045

**Table 7:** Distribution of the patients according to their kidney function and the viral load at the sixth month of the study

Variables	Moringa group		Control group		*P*
	*n* = 28	%	*n* = 29	%	
Creatinine (g/dl)					
Normal		89.3		75.9	0.18
Pathological		10.7		24.1	
VL (copies/ml)					
<1000		92.9		96.6	0.61
≥1000		7.1		4.4	

*VL* viral load

## Discussion

The M. O Lam. leaf powder supplementation intervention was effective for improving the patients’ body mass index and serum albumin levels compared to patients who only received nutritional counseling. In addition, as a secondary outcome, the intervention was effective for improving the BMI of patients with professional activity among those who were supplemented with M.O. Lam. leaf powder compared with those who received nutritional counseling. This BMI and serum albumin improvement with M. O Lam. leaf powder intervention associated with ART is an important finding and demonstrates the potential to improve the outcomes and health of PLHIV undergoing ART [35, 37, 38].

The results presented in this study on the nutrient contents of M. O Lam. leaf powder Congolese ecotype expand on the current literature regarding the potential use of M. O Lam. leaf powder as a dietary supplement for patients, especially those living in a limited resource setting. Recommendations for the use of M. O Lam. leaf powder to fight against malnutrition in limited resources settings have been advocated in literature [39–41]. In this study, we observed corroborative and consistent results about the amount of protein, the characteristics of the fat content, in which we listed unsaturated and saturated fatty acids, especially mono and polyunsaturated fatty acids, as well as the phytosterol and mineral or trace element content [18, 22, 39, 42–45]. Nonetheless, there is still a need for in vivo extended studies to better evaluate the impact of M.O. Lam. leaf powder protein in the human diet [44, 45].

The wide range of essential amino acids provided by the supplement might help to rebuild muscle mass tissue, fat mass, and restore bodily functions [18, 21, 46–48]. Several studies have attested the essential role of protein intake in the functioning of the organism in terms of the maintenance of body cells, increase in body weight, and a decrease in the viral load [35, 46, 47]. Our results indicated that M. O powder supplementation effect was more effective in the patients with professional activity (PA) than in those without PA. Beyond assets on psychological, economical even sociological on the patient wellness, the generated PA income, doubtless allowed patients in this study category to reach more easily the daily meals in which they mixed the powder of M.O. Houtzager review [49] on nutrition and HIV underlined that better socioeconomic status of patient might generate conditions on the psychological support nd way to access food securely. In summary, we recommend that an effort be continued to improve socio economical conditions of patients in limited-resources settings for a better HIV care outcomes. Although the BMI or biochemical parameters of patients in the CG were not significantly improved compared with the MG, nutritional counseling seemed to increase the patients’ knowledge about nutrition or the quality of their diet. Through this study, many patients discovered cheap and healthy diets in their environment. Indeed, nutritional counseling would improve nutritional knowledge and thereby improve dietary quality intake and reduce food insecurity [50, 51].

### Strengths and weaknesses of the study

In a limited resource setting, dietary supplementation with M. O Lam leaf powder, a locally produced food of plant origin with high nutritional values and accessible to all, has positive outcomes for PLHIV undergoing ART which might allow its integration into a nutrition program. Patients included in this study were motivated to participate and expected positive health changes; moreover, their motivation was boosted during the allocated time for the monthly supply of M.O. Lam. leaf powder and dietary advice.

However, some limitations have to be pointed out for this study: behavioral factors, important factors influencing weight, were difficult to measure in this type of study. The lack of quantification of the daily energy intake from meals (staple food) in which M. O Lam. leaf powder was mixed prevents us from drawing any conclusion about the real total daily energy intake of each patient. Despite the accessibility of M.O. to all, its use should remain under medical monitoring to ensure a safe level of consumption, notably due to the risk of high dietary protein intake in combination with antiretroviral drugs, which can aggravate pre-existing chronic renal insufficiencies and the racial predisposition for kidney damage [52–56]; renal function checkups must also be ensured. The absence or even a reduced effect of vegetable proteins on hemodynamics has been reported in the literature [53, 55], which might explain the absence of a significant statistical difference between the proportions of patients with higher than normal creatinemia between the study groups.

Other limitations of this study include a relatively small sample size and somewhat limited follow-up period. Finally, the compliance of M.O. Lam. leaf powder supplementation was checked monthly by the self-reporting of patients, by which most M. O use-related difficulties were recorded.

### Unanswered questions and future research

An unanswered question remains concerning the specific doses of M. O Lam. leaf powder required to improve the health and antiretroviral care outcomes of PLHIV early on in their HIV infection trajectory. Future trials should firstly assess the patient’s average daily energy intake before determining the quantity of M. O Lam. leaf powder to be consumed. Secondly, the comparison of the impact of supplementation might be observed through each subgroup of PLHIV in order to perceive the corresponding therapeutic doses.

Other unanswered questions concern the impact of M. O Lam. leaf powder supplementation on the patients’ immune system. This intervention was not effective at improving the lymphocyte counts or reducing the viral load of PLHIV undergoing ART. In this study, blood samples were taken on inception and in the sixth month, whereas we would recommend that future trials carry out immunovirological monitoring monthly to assess fluctuations that might be imputed to the intervention in each subgroup. Further trials should also include a larger sample size and a longer duration of follow-up to shed light on these remaining unanswered questions.

## Conclusions

The results of this study are encouraging, despite the limitations described above. They attest the positive role that nutritional support by M.O. Lam. leaf powder supplementation might have for PLHIV who are being treated with ART regimens in areas with limited resources. Supplementation with M.O. Lam. leaf powder can be used as an available local solution to not only ward off nutritional deficiencies but also boost and sustain a stable nutritional status of PLHIV on ART in limited resources settings. Nonetheless, this supplementation needs to be monitored by healthcare professionals, particularly in terms of the control of specific biological parameters, in order to obtain optimal results.

## Abbreviations

ACS/AMO CONGO: Action Communautaire Sida et Avenir Meilleur pour les Orphelins en République Démocratique du Congo
AIDS: Acquired immune deficiency syndrome
ART: Antiretroviral therapy
BMI: Body mass index
CD4: Cluster de differentiation 4
CG: Control group
DRC: Democratic Republic of the Congo
FFQ: Food Frequency Questionnaire
GATHER: Greet–Ask–Tell–Help–Explain–Return
HIV: Human immunodeficiency virus
INRB: Institut national de recherché Biomédicale
M.O.: *Moringa oleifera*
MG: Moringa intervention group
PLHIV: People living with HIV
PNLS: National Program for the Fight against AIDS (Programme national de lutte contre le sida)
SD: Standard deviation
UNIKIN: Université de Kinshasa
WHO: World Health Organization
